# Dynamical switching between different hippocampal rhythms

**DOI:** 10.1186/1471-2202-12-S1-P284

**Published:** 2011-07-18

**Authors:** Anastasia I Lavrova, Ekaterina A Zhuchkova, Susanne Schreiber, Lutz Schimansky-Geier

**Affiliations:** 1 Institute for Physics, Humboldt-Universität zu Berlin, Berlin, 12489, Germany; 2 Institute for Theoretical Biology, Humboldt-Universität zu Berlin, Berlin, 10115, Germany; 3 Bernstein Center for Computational Neuroscience, Berlin, 10115, Germany

## 

The hippocampal circuit can exhibit network oscillations in different frequency ranges (“gamma” - 30-80 Hz; “theta” - 4-12 Hz; as well as “theta/gamma” or a bursting regime) both *in vivo* and *in vitro* and switch between them [1]. These different oscillatory modes facilitate memory storage in the hippocampus and memory consolidation [2,3]. The hippocampal neuronal network consists of various types of connected cells that differ in morphology and functional properties, which allows them to provide oscillations with different periods, amplitudes, and phase shifts [1]. Dynamical switching between various rhythms is likely to depend on the local network structure of the neurons.

Our goal is to investigate how coupling strength and delayed propagation influence synchronization and switching between different oscillatory states in minimal neuronal networks. To this end, we constructed a simple model of neurons comprising two fast-spiking and two slow-spiking cells, respectively. Cells are synaptically connected in an all-to-all manner, with exception of the two slow-spiking cells. The network is described by coupled FitzHugh-Nagumo equations that well reproduce the dynamical behavior of different cells types: their periods, amplitudes, and phase shifts.

The model allows us to analyze the influence of synaptic strengths on the network synchronization and dynamical switching between theta, gamma, and bursting regimes. In particular, we perform a thorough bifurcation analysis and identify parameters of synaptic connections that can efficiently induce switches in the network activity. We show that depending on the coupling strengths between slow- and fast-spiking cells, abrupt changes between different rhythms can occur, similar to experimental observations.
